# Prediction of Acute Radiation Mucositis using an Oral Mucosal Dose Surface Model in Carbon Ion Radiotherapy for Head and Neck Tumors

**DOI:** 10.1371/journal.pone.0141734

**Published:** 2015-10-29

**Authors:** Atsushi Musha, Hirofumi Shimada, Katsuyuki Shirai, Jun-ichi Saitoh, Satoshi Yokoo, Kazuaki Chikamatsu, Tatsuya Ohno, Takashi Nakano

**Affiliations:** 1 Gunma University Heavy Ion Medical Center, Maebashi, Japan; 2 Department of Stomatology and Maxillofacial Surgery, Gunma University Graduate School of Medicine, Maebashi, Japan; 3 Department of Otolaryngology-Head and Neck Surgery, Gunma University Graduate School of Medicine, Maebashi, Japan; Georgetown University, UNITED STATES

## Abstract

**Purpose:**

To evaluate the dose-response relationship for development of acute radiation mucositis (ARM) using an oral mucosal dose surface model (OMDS-model) in carbon ion radiotherapy (C-ion RT) for head and neck tumors.

**Methods:**

Thirty-nine patients receiving C-ion RT for head and neck cancer were evaluated for ARM (once per week for 6 weeks) according to the Common Terminology Criteria for Adverse Events (CTCAE), version 4.0, and the Radiation Therapy Oncology Group (RTOG) scoring systems. The irradiation schedule typically used was 64 Gy [relative biological effectiveness (RBE)] in 16 fractions for 4 weeks. Maximum point doses in the palate and tongue were compared with ARM in each patient.

**Results:**

The location of the ARM coincided with the high-dose area in the OMDS-model. There was a clear dose-response relationship between maximum point dose and ARM grade assessed using the RTOG criteria but not the CTCAE. The threshold doses for grade 2–3 ARM in the palate and tongue were 43.0 Gy(RBE) and 54.3 Gy(RBE), respectively.

**Conclusions:**

The OMDS-model was useful for predicting the location and severity of ARM. Maximum point doses in the model correlated well with grade 2–3 ARM.

## Introduction

Acute radiation mucositis (ARM) usually occurs during or shortly after irradiation of patients with head and neck tumors. It impairs quality of life and decreases disease-fighting ability [1,2]. According to a systematic review, the incidence of ARM in patients with head and neck tumors receiving conventional radiotherapy is 97% overall and 34% for grade 3–4 tumors. In patients receiving chemoradiotherapy, it is 89% overall and 43% for grade 3–4 tumors [3].

Carbon ion radiotherapy (C-ion RT) provides a highly localized deposition of energy that can increase radiation doses to tumors while minimizing irradiation of adjacent normal tissues. With C-ion RT, tumor control is approximately 70–80% for locally advanced or postoperative recurrent non-squamous cell carcinomas of the head and neck, but only 11% for grade 3 or worse tumors accompanied by ARM according to the Radiation Therapy Oncology Group (RTOG) scoring system [4–8]. Since C-ion RT results in a steep dose gradient around the target, it potentially minimizes high dose volumes to the mucosa that may cause high-grade ARM. However, the relationship between radiation dose and development of ARM in C-ion RT is unclear.

We developed an oral mucosal dose surface model (OMDS-model) using three-dimensional (3D) treatment planning data. The purpose of the present study was to determine whether the point dose and dose-volume histogram (DVH) parameters obtained in the OMDS-model correlate with the severity of ARM, and whether the model is a useful tool for predicting ARM in patients with head and neck tumors treated via C-ion RT.

## Materials and Methods

### Patient characteristics

Between 2011 and 2012, 39 patients with head and neck tumors were treated via C-ion RT at the Gunma University Heavy Ion Medical Center. The protocol for C-ion RT was approved by the Institutional Review Board of Gunma University. In our hospital, the C-ion RT protocol is indicated for X-ray resistant non-squamous cell carcinomas. Written informed consent was obtained from all patients before treatment. Patient and tumor characteristics are summarized in Table 1. Primary tumors were located in the nasal cavity (*n* = 21), parotid gland (*n* = 4), pharynx (*n* = 3), maxilla (*n* = 3), and root of the tongue, buccal region, external auditory canal, auricular region, or maxillary sinus (*n* = 8).

**Table 1:** Patient (n = 39) and tumor characteristics, in patients with head and neck tumors treated via carbon ion radiotherapy.

Age (years)	64 (39–91)
Sex	
Male	16 (41)
Female	23 (59)
Histological type	
Malignant melanoma	18 (46)
Adenoid cystic carcinoma	11 (28)
Mucoepidermoid carcinoma	3 (8)
Others	7 (18)
Region	
Nasal cavity	21 (54)
Parotid gland	4 (10)
Pharynx	3 (8)
Maxilla	3 (8)
Others	8 (20)
Total dose	
57.6 Gy(RBE)	5 (13)
64.0 Gy(RBE)	32 (82)
70.4 Gy(RBE)	2 (5)

Data are reported as mean (range) or n (%).

RBE, relative biological effectiveness

### Carbon ion radiotherapy

The patients were positioned in customized cradles (Moldcare, Alcare, Tokyo, Japan) and immobilized by use of a thermoplastic shell (Shellfitter, Kuraray, Osaka, Japan). A customized mouthpiece was used to fix the teeth of both jaws and to maintain the position of the lower jaw. Computed tomography (CT) images with a 2-mm thickness were acquired for treatment planning. Magnetic resonance images served as a reference for planning CT. A margin of at least 5 mm was usually added to the gross tumor volume (GTV) to define the clinical target volume (CTV); if the GTV was very close to or invaded the organs at risk (OARs), the margin was accordingly modified. OARs (eye, optic nerve, optic chiasm, inner ear, brain stem, spinal cord, mandible, palate, and tongue) were outlined on the planning CT scan for treatment planning and DVH analysis. To account for patient setup error, a 2-mm margin was added to the CTV (planning tumor volume). Treatment planning was performed using an XiO-N system (Elekta AB, Stockholm, Sweden).

Physical dose calculations were performed via a pencil beam algorithm. The clinical dose distribution was calculated according to the physical dose and the relative biological effectiveness (RBE). The dose of C-ion RT was expressed as “Gy(RBE)” [physical carbon ion dose (Gy) × RBE]. The number of fractions was 16, and the overall treatment time was 4 weeks (4 fractions per week). In accordance with the clinical protocol, 32 patients received 64.0 Gy(RBE) in 16 fractions, five patients received 57.6 Gy(RBE) (in these patients, the skin or mucosa was considered to be widely irradiated), and two sarcoma patients received 70.4 Gy(RBE). Concomitant chemotherapy (Day 1: 120 mg/m^2^ dacarbazine, 70 mg/m^2^ nimustine, and 0.7 mg/m^2^ vincristine; Day 2–5: 120 mg/m^2^ dacarbazine) consisting of three courses at 4-week intervals was administered to 11 patients with malignant melanomas, as follows: the first course at the start of C-ion RT, the second course after completion of C-ion RT, and one course thereafter.

### Assessment of acute radiation mucositis

Mucosal reactions were assessed, and oral care was performed by a dental hygienist at least once per week before, during, and for 2 weeks after C-ion RT for a total duration of 6 weeks. Temporal mucosal reactions of the palate and tongue were photographed by well-trained physicians. ARM was graded weekly in each patient according to the Common Terminology Criteria for Adverse Events (CTCAE), version 4.0 [9] and the RTOG grading system. The highest grade of ARM in each toxicity assessment was used in the analyses.

### Oral mucosal dose surface model and dose-volume histogram

**Fig 1:**
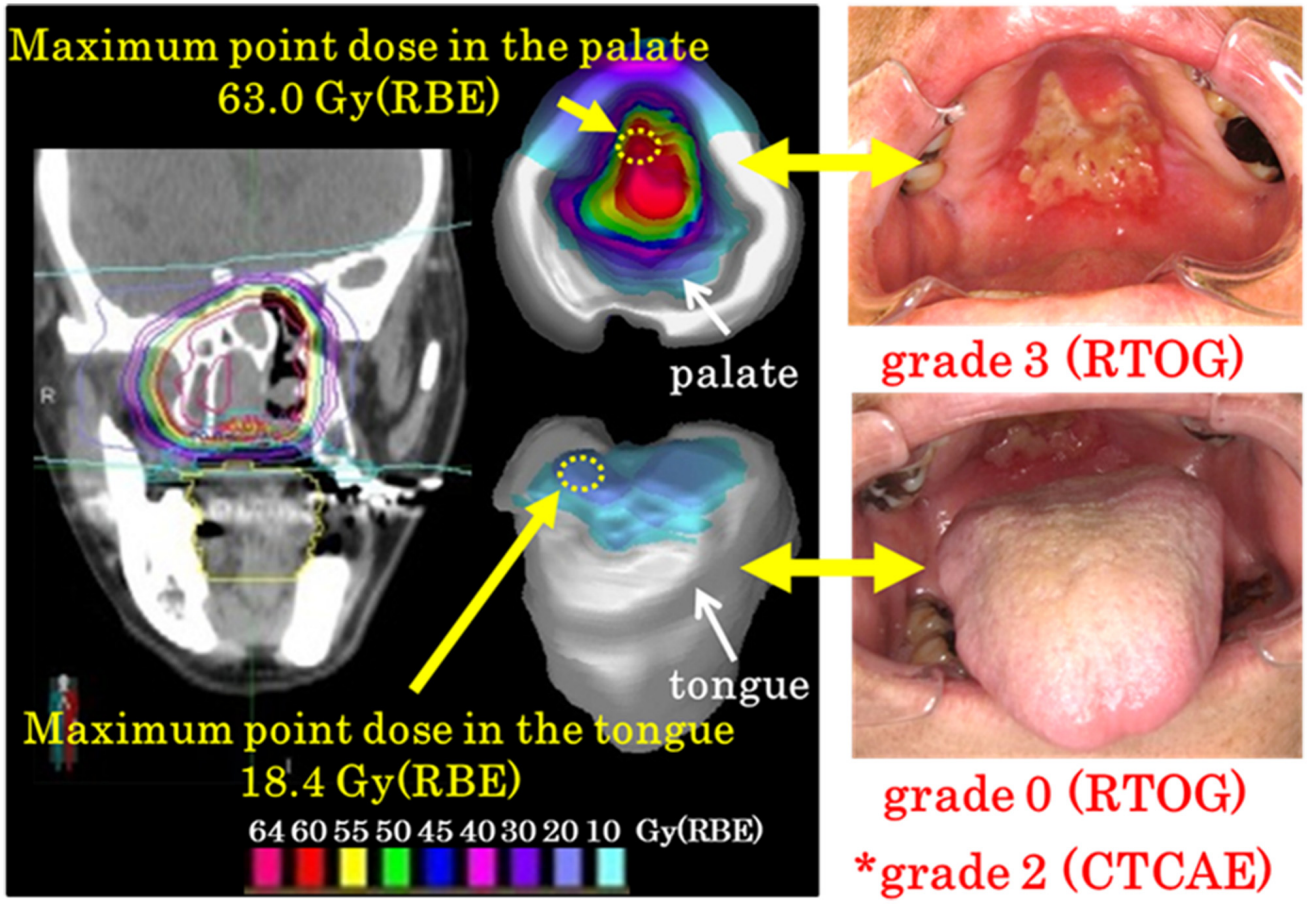
Oral mucosal dose surface model and corresponding mucositis. Representative dose distributions (coronal image, palate, and tongue surface) in a patient with malignant melanoma of the right nasal cavity are shown. The location of the acute radiation mucositis (ARM) approximated the high radiation dose area. ARM was classified as grade 2 according to version 4 of the Common Terminology Criteria for Adverse Events (CTCAE)* and as grade 3 (palate) and grade 0 (tongue) according to the Radiation Therapy Oncology Group (RTOG) criteria. RBE, relative biological effectiveness.

Commercially available software (MIM Maestro version 6.0.2.) was used to create OMDS-models of the tongue and palate. We exported a plan for MIM from the XiO-N system and displayed the 3D dose distribution on the surface of the palate and tongue. The locations of the high-dose area in the OMDS-model and the high-grade ARM were compared during and shortly after C-ion RT (Fig 1).

Maximum point doses for the palate and tongue were identified in each patient, and the relationship between doses and ARM severity was determined.

DVHs of ARM in the tongue were analyzed using the MIM Maestro software. Contouring was done from the top of the tongue to the lower border of the mandible on each CT slice. V*n* [total tongue volume irradiated at >*n* Gy(RBE), with *n* ranging from 0 to 60 in increments of 5] was calculated. Vn values were compared between patients with grade 0–1 and grade 2–3 ARM. Eight cases of ARM were excluded from the analysis of the tongue owing to metal artifacts on the lower jaw in planning CT scans. DVH evaluation of the palate was not performed because it was difficult to identify the palate volume on CT images. We also show the temporal pattern of ARM according to the mucositis grade.

### Statistical analysis

Results are expressed as mean ± standard deviation (SD) or standard error (SE) of the mean. Statistical differences were determined using a two-sided Student’s *t* test. An independent *t* test was used to compare differences in the maximum point dose between grade 0–1 and grade 2–3 ARM. Receiver operating characteristic (ROC) curves were generated to predict the dose at the onset of ARM, and the data were analyzed using SPSS Statistics 21 software (IBM Corp, Armonk, NY). Differences with a *p* value <0.05 were considered significant.

## Results

### Incidence of acute radiation mucositis

Table 2 shows the locations and grades of ARM in the 39 patients in our study. There were no cases of grade 4 ARM. The incidence of grade 2–3 ARM was statistically similar regardless of the criteria used for grading (RTOG, 85%; CTCAE, 56%, *p* = 0.681). Grade 2–3 ARM was significantly more frequent in the palate than the tongue (82% versus 31% for cases graded according to the RTOG criteria, *p* = 0.015). ARM occurred for 94% of the patients who underwent combination therapy with C-ion RT and DAV and 85% of the patients who underwent C-ion RT only (*p* = 0.803).

**Table 2:** Locations and stages of acute radiation mucositis in patients with head and neck tumors treated via carbon ion radiotherapy.

	Grade 0	Grade 1	Grade 2	Grade 3	Grade 4
CTCAE (palate/tongue)	4	13	18	4	0
RTOG (palate/tongue)	6	0	19	14	0
RTOG (palate)	7	0	19	13	0
RTOG (tongue)	20	7	8	4	0

CTCAE = Common Terminology Criteria for Adverse Events; RTOG = Radiation Therapy Oncology Group

### Relationship between the maximum point dose and acute radiation mucositis graded according to the Common Terminology Criteria for Adverse Events

**Fig 2:**
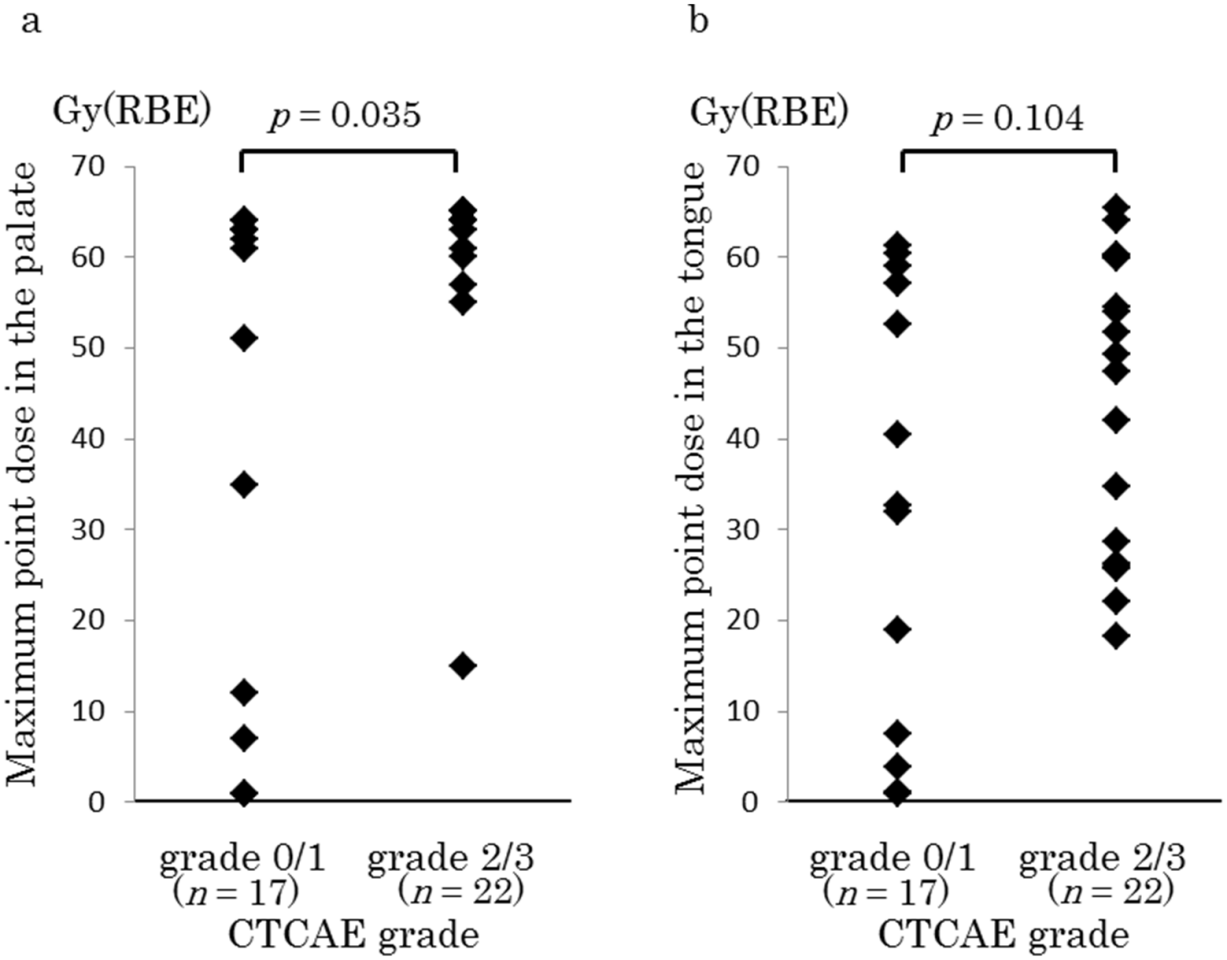
Relationship between the maximum point dose in the oral mucosal dose surface model and acute radiation mucositis (ARM) grade (Common Terminology Criteria for Adverse Events [CTCAE] classification). Maximum doses in the palate (a) and tongue (b) for grade 0–1 and grade 2–3 ARM. Data are presented as mean ± standard deviation. RBE, relative biological effectiveness.

The maximum point doses in the palate were 42.1 ± 26.9 and 60.4 ± 11.3 Gy(RBE) for grades 0–1 and 2–3, respectively; this difference was significant (*p* = 0.035, Fig 2a). The maximum point doses in the tongue were 32.9 ± 24.1 and 44.8 ± 16.1 Gy(RBE) for grades 0–1 and 2–3, respectively; these doses did not differ significantly (*p* = 0.104, Fig 2b).

### Relationship between the maximum point dose and acute radiation mucositis graded according to the Radiation Therapy Oncology Group criteria

**Fig 3:**
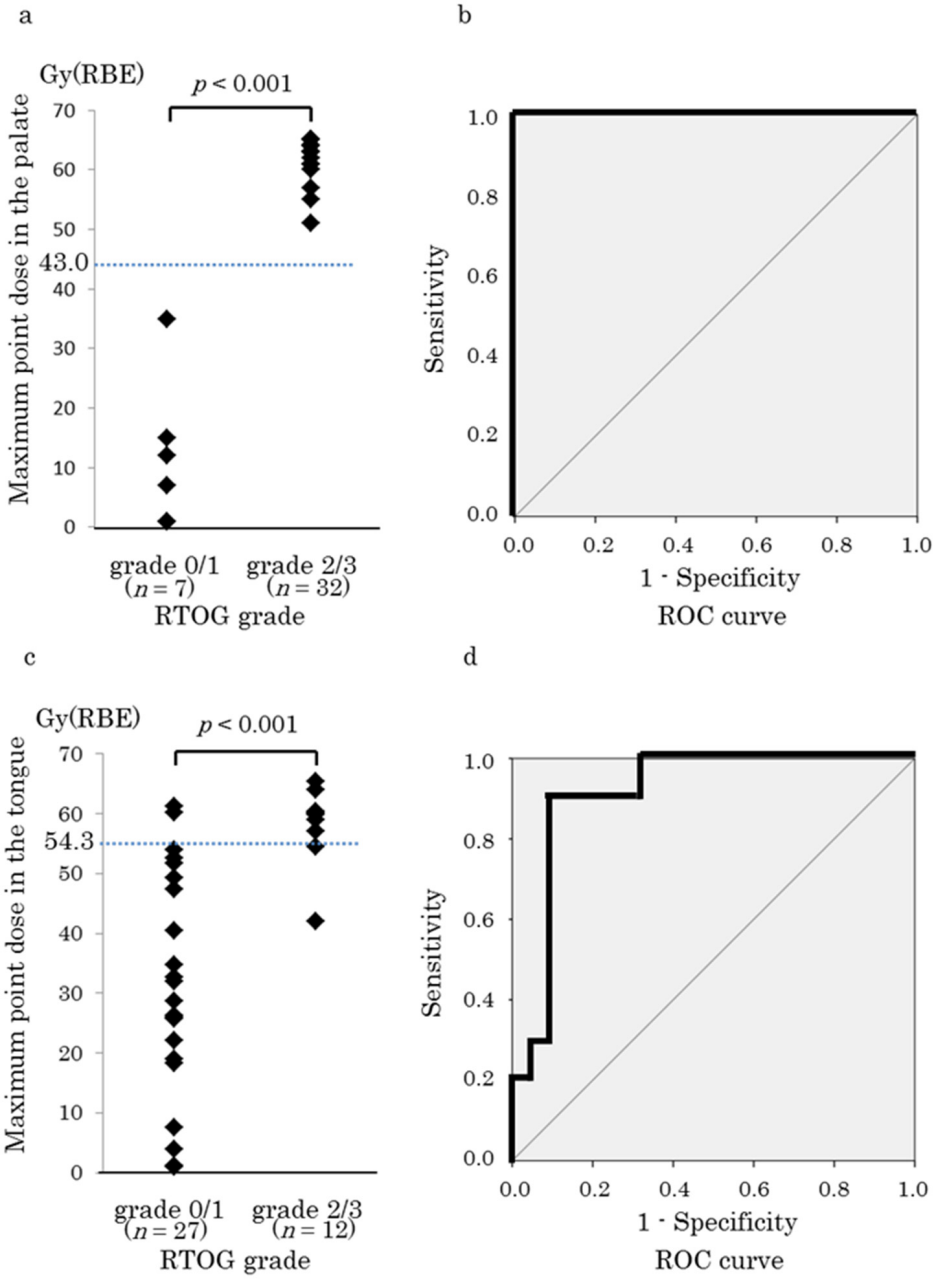
Relationship between the maximum point dose in the oral mucosal dose surface model and acute radiation mucositis (ARM) grade (Radiation Therapy Oncology Group [RTOG] classification). (a) Maximum doses in the palate for grade 0–1 and grade 2–3 ARM. (b) Receiver operating characteristics (ROC) curve for grade 2–3 ARM in the palate (sensitivity, 1.0; specificity, 1.0). (c) Maximum doses in the tongue for grade 0–1 and grade 2–3 ARM. (d) ROC curve for grade 2–3 ARM in the tongue (sensitivity, 0.9; specificity, 0.9). Data are presented as mean ± standard deviation. RBE, relative biological effectiveness.

The maximum point doses in the palate were 11.8 ± 12.7 and 62.4 ± 3.3 Gy(RBE) for grades 0–1 and 2–3, respectively; this difference was significant (*p* < 0.001, Fig 3a). In the ROC curve, the cutoff value was 43.0 Gy(RBE) for RTOG grade 2–3 ARM in the palate (sensitivity, 1.0; specificity, 1.0; Fig 3b).

The maximum point doses in the tongue were 31.6 ± 18.8 and 58.3 ± 6.5 Gy(RBE) for grades 0–1 and 2–3; respectively; this difference was significant (*p* < 0.001, Fig 3c). The cutoff value was 54.3 Gy(RBE) for RTOG grade 2–3 ARM in the tongue (sensitivity, 0.9; specificity, 0.9; Fig 3d).

### Comparison of the Vn values for acute radiation mucositis in the tongue

**Fig 4:**
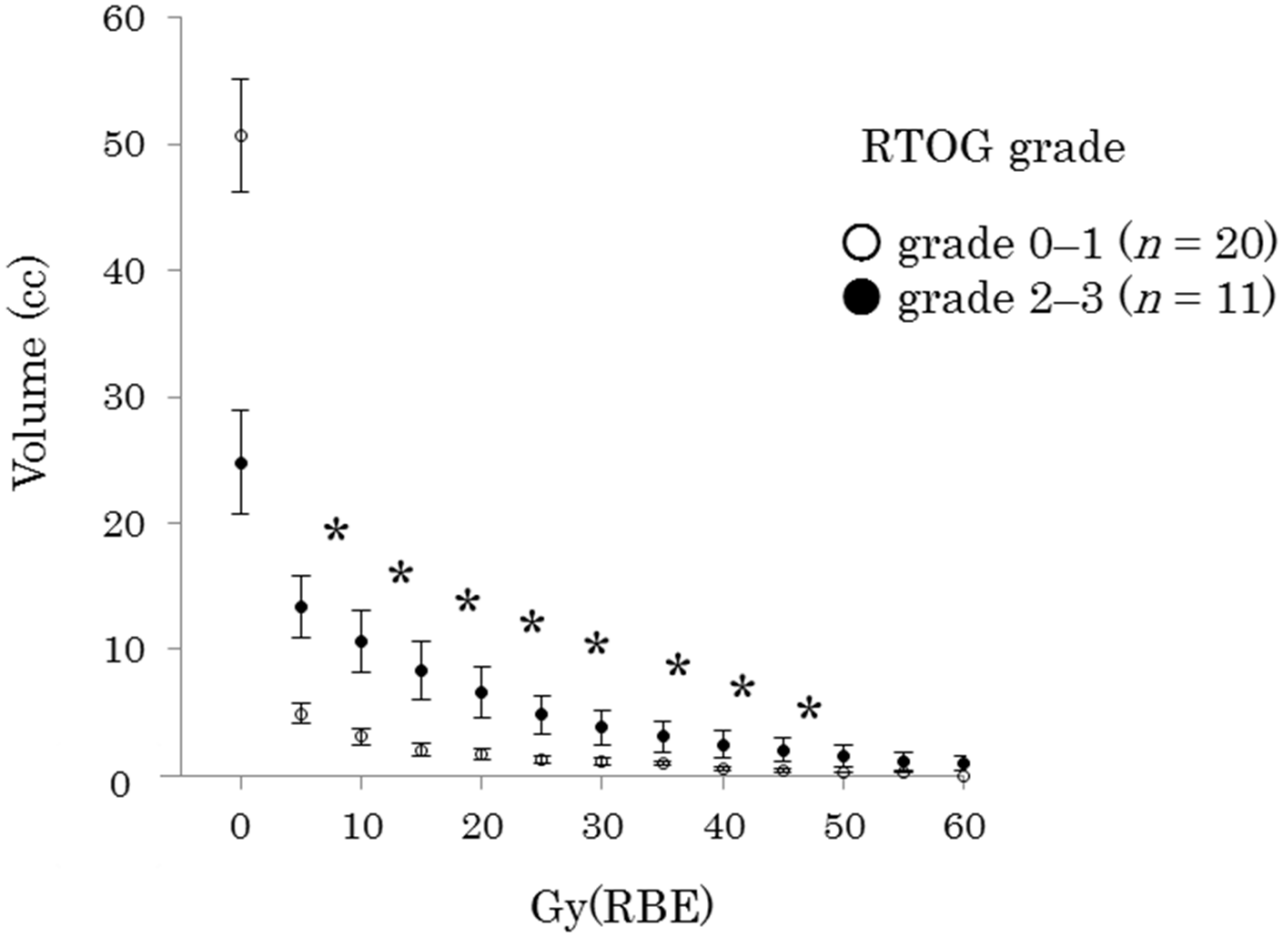
Comparison of a dose-volume histogram of the tongue with acute radiation mucositis (ARM) grade. ARM was graded according to the Radiation Therapy Oncology Group (RTOG) criteria. Data are presented as mean ± standard error. *p < 0.05. RBE, relative biological effectiveness.

Fig 4 compares the DVHs for the tongue between ARM grades 0–1 and 2–3. The V5–V40 was significantly lower for grade 0–1 than for grade 2–3.

### Temporal pattern of acute radiation mucositis according to mucositis grade

**Fig 5:**
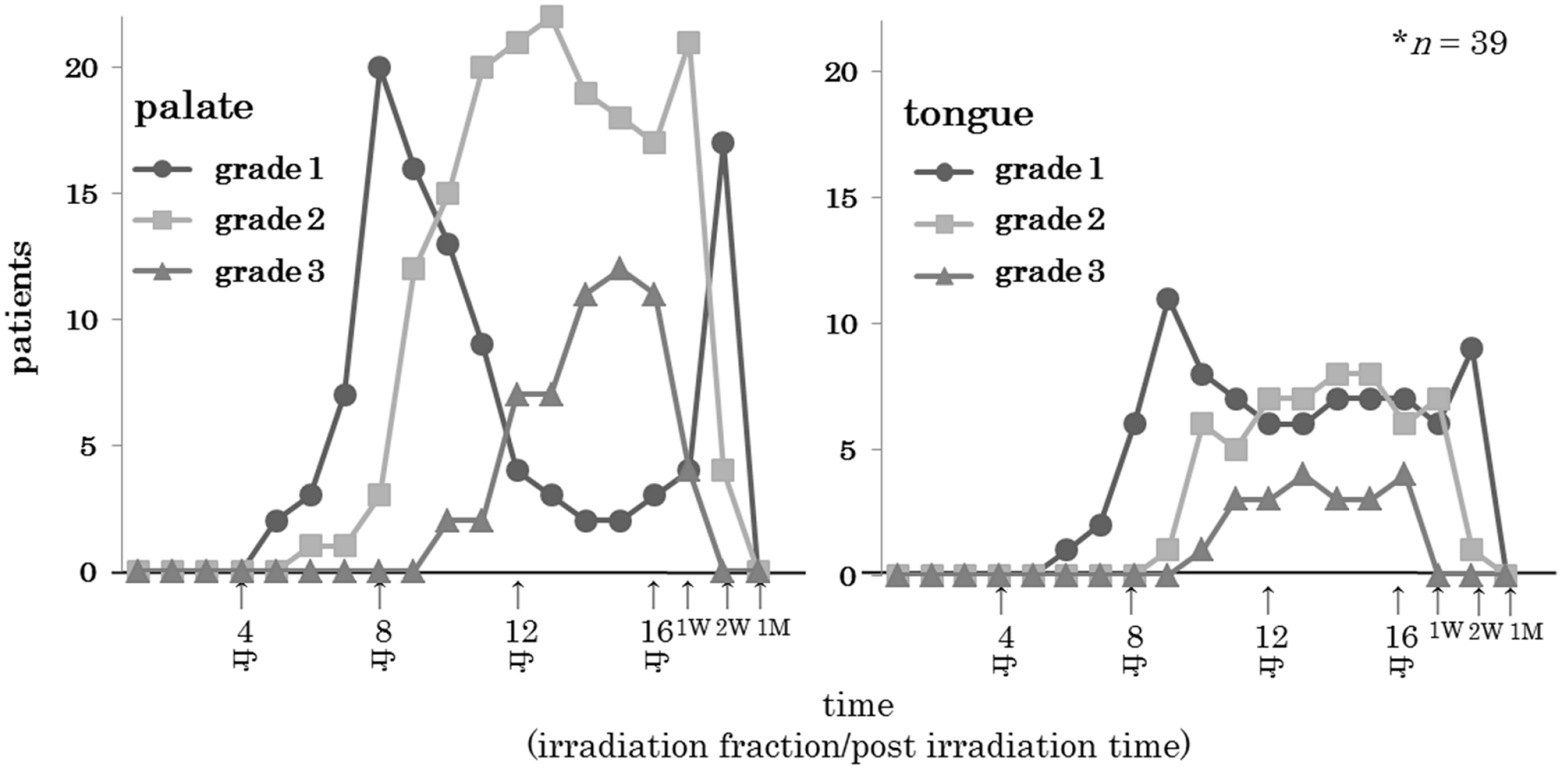
Temporal pattern of acute radiation mucositis (ARM) of the palate or tongue. We assessed the change in ARM over time. The times are indicated as fractions 1 to 16, and 1 or 2 weeks and 1 month post irradiation.

The RTOG grade for ARM in the palate and tongue gradually increased over an approximate one-week period after initiation of the C-ion RT. The ARM grade in the palate was 2 times higher than that in the tongue. Following 16 irradiation procedures, the ARM grade gradually decreased over a one-month period. The ARM recovery period did not differ significantly based on C-ion RT dose. When the ARM healed, dietary intake was restored (Fig 5).

## Discussion

In the present study, we developed an OMDS-model to evaluate the point doses and DVH parameters associated with ARM in patients with head and neck tumors treated via C-ion RT. The location of the ARM coincided with the high-dose area in this model. A maximum point dose of 43.0 Gy(RBE) in the palate and 54.3 Gy(RBE) in the tongue predicted the development of grade ≥2 ARM as evaluated using the RTOG criteria (Fig 3). Significant differences were not present for the radiation dose or the healing period (Fig 5). ARM rapidly improved after C-ion RT, and none of the patients experienced treatment-related death or impaired quality of life.

In the OMDS-model, there was a clear dose-response relationship between maximum point dose and ARM grade assessed using the RTOG criteria but not the CTCAE. This suggests that the RTOG criteria reflect the dose dependency of the response whereas the CTCAE do not. The CTCAE (version 4.0) for ARM include pain thresholds and changes in dietary intake [9], whereas the RTOG criteria are more objective (e.g., extent of mucosal redness and ulceration). Although some of our patients presented with grade 2 or 3 ARM in highly localized areas, their dietary intake was largely unaffected. We believe that the location of the primary tumor and the steep gradient of the dose distribution in C-ion RT alleviate the difficulty with food intake in patients with head and neck tumors.

ARM was relatively milder in the tongue than the palate when evaluated using the RTOG criteria. There are three possible reasons for this. First, the most common site of primary head and neck tumors is the nasal cavity. Since C-ion RT has a very steep penumbra, the thickness of the mouthpiece (5 mm) may have significantly reduced the dose in the tongue (Fig 1). Second, the tolerance dose may be higher in the tongue than the palate. In the present study, the threshold dose for development of grade ≥2 ARM was higher in the tongue than the palate. Third, the tongue is muscle-rich and well supplied with blood, which facilitates its recovery from radiation damage.

A DVH showed a significant difference between the development of grade 2–3 versus grade 0–1 ARM in the tongue at low to medium radiation doses (V5–V40) (Fig 4). Sanguineti et al. [10] found that exposure of oral mucosa to 10.1 Gy/week predicted the occurrence of mucositis during intensity-modulated radiation therapy (IMRT). Others showed that point doses up to 32 Gy in the oral cavity correlated with grade 2 mucositis evaluated using the CTCAE [11]. These results and ours suggest that low to medium, as well as high, radiation dose volumes in the oral mucosa may promote grade 2–3 ARM development. Further research is required to identify the DVH parameters associated with ARM.

Our study had three limitations. First, we excluded the palate from DVH analysis because its volume on CT images could not be identified. Second, we only evaluated ARM. Late adverse effects in the oral mucosa should be examined in the future, because some studies suggest a correlation between the severity of acute and late toxicities [12–14]. Third, whether the OMDS-model is applicable to other irradiation modalities, such as conventional 3D conformal radiotherapy, IMRT, and proton therapy, is unclear.

In conclusion, the OMDS-model was useful for predicting the location and severity of ARM. The maximum point dose in this model correlated well with grade 2–3 ARM. Since severe mucositis worsens quality of life and interferes with chemotherapy and radiotherapy [1,15], we recommend that patients and the medical team (radiation oncologists, dentists, dental hygienists, nurses, nutritionists, radiotherapists, and medical physicians) share information about the onset area and severity of ARM using the OMDS-model to facilitate early intervention for oral care.
